# Gait characteristics associated with the foot and ankle in inflammatory arthritis: a systematic review and meta-analysis

**DOI:** 10.1186/1757-1146-8-S2-P2

**Published:** 2015-09-22

**Authors:** Matthew Carroll, Priya Parmar, Nicola Dalbeth, Mark Boocock, Keith Rome

**Affiliations:** 1 Department of Podiatry, Auckland University of Technology, Health & Rehabilitation Research Institute, Auckland, 0627, New Zealand; 2 Auckland University of Technology, National Institute for Stroke and Applied Neurosciences, Auckland, New Zealand; 3 Department of Medicine, Auckland University, Auckland, New Zealand; 4 Department of Physiotherapy, Auckland University of Technology, Health & Rehabilitation Research Institute, Auckland, 0627, New Zealand

**Keywords:** Gait, Rheumatoid arthritis, Ankylosing spondylitis, Psoriatic arthritis, Gout

## Background

Gait analysis is increasingly being used to characterise dysfunction of the lower limb and foot in people with inflammatory arthritis (IA). The aim of the systematic review was to evaluate the spatiotemporal, foot and ankle kinematic, kinetic, peak plantar pressure and muscle activity parameters between patients with inflammatory arthritis and healthy controls.

## Methods

An electronic literature search was performed on Medline, CINAHL, SportsDiscus and The Cochrane Library. Methodological quality was assessed using a modified Quality Index. Effect sizes with 95% confidence intervals (CI) were calculated as the standardised mean difference (SMD). Meta-analysis was conducted if studies were homogenous.

## Results

Thirty six studies with quality ranging from high to low met the inclusion criteria. The majority of studies reported gait parameters in RA. The gait pattern in RA was characterised by decreased walking speed (SMD 95% CI -1.57, -2.25 to -0.89), decreased cadence (SMD -0.97, -1.49 to -0.45), decreased stride length (SMD -1.66, -1.84 to -1.49), decreased ankle power (SMD -1.36, -1.70 to -1.02), increased double limb support time (SMD 1.03, 0.84 to 1.22), and peak plantar pressures at the forefoot (SMD 1.11, 0.76 to 1.45). Walking velocity was reduced in psoriatic arthritis and gout with no differences in ankylosing spondylitis. No studies have been conducted in polymyalgia rheumatica, systemic sclerosis or systemic lupus erythematosus.

## Conclusions

The review identified the majority of studies reporting gait adaptations in RA, but limited evidence relating to other IA conditions. Poor data reporting, small sample sizes and heterogeneity across IA conditions limit the interpretation of the findings. Future studies may consider a standardised analytical approach to gait analysis that will provide clinicians and researchers with objective evidence of foot function in people with IA.

